# Compound heterozygous mutations in glycyl-tRNA synthetase (*GARS*) cause mitochondrial respiratory chain dysfunction

**DOI:** 10.1371/journal.pone.0178125

**Published:** 2017-06-08

**Authors:** Michael Nafisinia, Lisa G. Riley, Wendy A. Gold, Kaustuv Bhattacharya, Carolyn R. Broderick, David R. Thorburn, Cas Simons, John Christodoulou

**Affiliations:** 1 Genetic Metabolic Disorders Research Unit, Western Sydney Genetics Program, The Children’s Hospital at Westmead, Sydney, New South Wales, Australia; 2 Discipline of Child & Adolescent Health, Sydney Medical School, University of Sydney, Sydney, New South Wales, Australia; 3 Discipline of Genetic Medicine, Sydney Medical School, University of Sydney, Sydney, New South Wales, Australia; 4 Genetic Metabolic Disorders Service, Western Sydney Genetics Program, The Children’s Hospital at Westmead, Sydney, New South Wales, Australia; 5 Children’s Hospital Institute of Sports Medicine, The Children’s Hospital at Westmead, Sydney, New South Wales, Australia; 6 School of Medical Sciences, UNSW, Sydney, New South Wales, Australia; 7 Murdoch Childrens Research Institute and Victorian Clinical Genetics Services, Royal Children’s Hospital, and Department of Paediatrics, University of Melbourne, Melbourne, Victoria, Australia; 8 Institute for Molecular Bioscience, The University of Queensland, St Lucia, Queensland, Australia; Texas Technical University Health Sciences Center, UNITED STATES

## Abstract

Glycyl-tRNA synthetase (*GARS*; OMIM 600287) is one of thirty-seven tRNA-synthetase genes that catalyses the synthesis of glycyl-tRNA, which is required to insert glycine into proteins within the cytosol and mitochondria. To date, eighteen mutations in *GARS* have been reported in patients with autosomal-dominant Charcot-Marie-Tooth disease type 2D (CMT2D; OMIM 601472), and/or distal spinal muscular atrophy type V (dSMA-V; OMIM 600794). In this study, we report a patient with clinical and biochemical features suggestive of a mitochondrial respiratory chain (MRC) disorder including mild left ventricular posterior wall hypertrophy, exercise intolerance, and lactic acidosis. Using whole exome sequencing we identified compound heterozygous novel variants, c.803C>T; p.(Thr268Ile) and c.1234C>T; p.(Arg412Cys), in *GARS* in the proband. Spectrophotometric evaluation of the MRC complexes showed reduced activity of Complex I, III and IV in patient skeletal muscle and reduced Complex I and IV activity in the patient liver, with Complex IV being the most severely affected in both tissues. Immunoblot analysis of GARS protein and subunits of the MRC enzyme complexes in patient fibroblast extracts showed significant reduction in GARS protein levels and Complex IV. Together these studies provide evidence that the identified compound heterozygous *GARS* variants may be the cause of the mitochondrial dysfunction in our patient.

## Introduction

Aminoacyl-tRNA synthetases (ARS) are ubiquitously expressed essential enzymes responsible for attaching amino acid residues to their cognate tRNA molecules, which is the first step of protein translation in the cytoplasm and mitochondria [1]. Human ARS proteins consist of three groups: cytoplasmic, mitochondrial, and bifunctional. For most ARS, the cytoplasmic and mitochondrial ARS for each amino acid are encoded by distinctly different nuclear genes. However, *GARS* (OMIM: 601472) and *KARS* (OMIM: 613641) encode both the cytoplasmic and mitochondrial ARS [2]. It is noteworthy that nuclear genes encode the mitochondrial ARSs that are then imported into mitochondria [2].

The cytoplasmic and mitochondrial isoforms of glycyl-tRNA synthetase, encoded by *GARS*, differ by a 54 amino acid N-terminal mitochondrial targeting sequence [3,4]. Mitochondrial GARS is required for the synthesis of 13 subunits of the MRC complexes, including subunits of Complex I, Complex III, Complex IV and Complex V [2]. The human GARS belongs to the class IIA aminoacyl- tRNA synthetases, with the cytosolic isoform having 685 amino acids and the mitochondrial isoform having 739 amino acids [4]. Both isoforms include the N-terminal WHEP-TRS domain composed of the amino acid residues 62–122, a catalytic domain (124–608), and a C terminal anticodon-binding domain (602–726) [4].

Missense mutations in *GARS* were first described in 2003 in association with Charcot-Marie-Tooth disease type 2D (CMT2D: OMIM 601472), and distal Spinal Muscular Atrophy type V (dSMA-V; OMIM 600794) in five families with atrophy and weakness of the hand muscles [5]. Several reports have since been published detailing pathogenic *GARS* missense mutations, mostly autosomal dominant, but some *de novo* [1,5–12] (Table 1).

**Table 1:** Clinical phenotypes associated with *GARS* variants in human.

	Kawakami et al. (2014)	Del Bo et al. (2006)	Antonellis et al. (2003)	Rohkamm et al. (2007)	Sivakumar et al. (2005) Antonellis et al. (2003)	Sivakumar et al. (2005) Antonellis et al. (2003)	Lee et al. (2012)	Lee et al. (2012)	Liao et al. (2015)	Liao et al. (2015)	Sivakumar et al. (2005) Antonellis et al. (2003)	Hamaguchi et al. (2010)	James et al. (2006)	James et al. (2006)	James et al. (2006)	Klein et al. (2014)	Sivakumar et al. (2005)	Dubourg et al. (2006)	McMillan at al. (2014)	This Study
*GARS* variants	c.815T>A p.Leu218Gln	c.2016G>A p. Asp500Asn	2094G>C p. Gly526Arg	c.688 C > T p.Ala57Val	c.730A>G p.Glu71Gly	c.904C>T p.Leu129Pro	c.598G>A p. Asp200Asn	c.794C>T p. Ser265Phe	c.598G>T p. Asp146Tyr	c.875T>G p.Met238Arg	c.1236G>C p.Gly240Arg	c.893C>T p. Pro244Leu	c.1358A>T p.Ile280Phe	c.2313G>C p.Gly598Ala	c.2260C>T p. Ser581Leu	p.Ile334Phe	p.His418Arg	c.2094 A>G p.Gly526Arg	c.1904C > T p.Ser635Leu c.1787G > A p.Arg596Gln	c.803C>T p.Thr268Ile c.1234C>T p.Arg412Cys
Inheritance	AD	AD	AD	AD	AD	AD	AD	AD	*De novo*	*De novo*	AD	ND	AD	*De novo*	AD	AD	AD	AD	AR	AR
Protein domain potentially affected	CD	Ins III	CD	WD	CD	CD	Ins I	Ins III	Ins I	CD	CD	CD	CD	ACBD	ACBD	Ins II	CD	CD	ACBD	CD
Phenotype	CMT2	CMT2D/ dSMA-V	dSMA-V	dHMN-V	CMT2D/ dSMA-V	dSMA-V	dHMN- V	dHMN- V	CMT2	CMT2	CMT2D	CMT2	dHMN- V	Infantile SMA	CMT2	CMT2	dSMA-V	dHMN-V	MRCD	MRCD
Cohort	1 affected	4 affected	2 affected	1 affected	17 affected	5 affected	1 affected	1 affected	1 affected	1 affected	14 affected	1 affected	1 affected	1 affected	1 affected	1 affected	1 affected	16 affected	1 affected	1 affected
Age at onset	<2 Years	10–35 Years (average)	13–26 Years (average)	12 Years	18 Years (average)	16.9 Years (average)	15Years	13Years	<6 months	2 Years	23 Years (average)	10 Years	11Years	<6 m	27 Years	24 Years	26Years (average)	23.3Years (average)	7 Years	6 Years
Consanguinity	NS	NS	NS	NS	NS	NS	NS	NS	NS	NS	NS	No	NS	NS	NS	NS	NS	No	No	No
Ethnicity	Japan	Italy	Sephardic Jewish	Ghana	Mongolia	Bulgaria	Korea	Korea	Taiwan	Taiwan	North America	Japan	UK	UK	UK	USA	UK/Australia	France	ND	UK/Australia
Clinical features	delayed onset of walking, slow running	atrophy and weakness of the hand muscles	atrophy and weakness of the hand muscles	atrophy and weakness of the hand muscles	atrophy and weakness of the hand muscles	atrophy and weakness of the hand muscles	atrophy and weakness of the hand muscles	atrophy and weakness of the hand muscles	delayed milestones, severe generalised weakness	delayed onset of walking, unsteady gait	atrophy and weakness of the hand muscles	slow running	distal limb muscle atrophy and weakness	delayed milestones, severe generalised weakness	distal limb muscle weakness	progressive unsteadiness walking	atrophy and weakness of the hand muscles	atrophy and weakness of the hand muscles or distal four limbs	leukoencephalopathy, lactic acidosis and myalgia	fatigue, exercise-lactic acidosis, mild cardiomyopathy
Functional studies	ND	ND	ND	ND	ND	ND	ND	ND	ND	ND	ND	ND	ND	ND	ND	ND	ND	ND	ND	MRC enzymology, immunoblotting for *GARS* protein and subunits for MRC enzyme complexes

ND = not done; NS–not stated; CD: Catalytic domain, DI: dimer interface, WD: WHEP domain, Ins I: Insertion I domain, Ins II: Insertion II domain, Ins III: Insertion III domain, ACBD: anticodon binding domain; AD: autosomal dominant, AR: autosomal recessive; CMT2: Charcot-Marie-Tooth hereditary neuropathy type 2; CMT2D: upper limb predominant CMT2; SMA: Spinal muscular atrophy; dSMA: distal SMA, dHMN: distal hereditary motor neuropathy type V, MRC: Mitochondrial respiratory chain, MRCD: Mitochondrial respiratory chain disorder.

However, McMillan and colleagues reported compound heterozygous variants in *GARS* for the first time, in a 12-year old girl with clinical manifestations suggestive of mitochondrial disease, including exercise-induced myalgia, non-compaction cardiomyopathy, lactic acidaemia, and abnormal T2 and T2 FLAIR hyperintensities in the periventricular and trigonal white matter bilaterally on magnetic resonance imaging (MRI)[13]. Herewith, we report the identification of compound heterozygous variants in the *GARS* gene in a 16 year-old female who presented with exercise-intolerance, mild cardiomyopathy and lactic acidosis. Also, our patient had clear evidence of a MRC enzyme deficiency, supporting an expansion of the phenotypic spectrum associated with *GARS* mutations to include mitochondrial disease in cases of autosomal recessive inheritance.

## Material and methods

### Patient clinical summary

The proband is a 16 year-old female of non-consanguineous Caucasian origin of British descent. She is the elder of two siblings with each parent being of elite athletic calibre (one represented their country in the Olympics). Difficulty with exercise was first noted at the age of 6 years when the proband started vomiting and became pale and lethargic on a cross-country walk. An echocardiogram and a 24 Holter assessment at the age of 9 years were normal. At this time, she completed isotonic exercise tests using the standard Bruce protocol, completing 8 minutes and 35 seconds, stopping due to breathlessness and a “sore chest.” She reached her anaerobic threshold early at 2.03 minutes with VO_2_ max being 57% of predicted. At 11 years of age, the Bruce exercise test was repeated by an exercise physiologist. She fatigued at 6.12 minutes having a VO_2_ max of 16.8 ml/kg/min (37% of predicted), with a very high respiratory exchange ratio (VCO_2_ /VO_2_) of 1.6.

The respiratory exchange ratio increases with increasing exercise intensity and is an indirect reflection of proportion of energy derived from carbohydrate metabolism vs. lipid oxidation. The high respiratory exchange ratio was consistent with CHO metabolism as the predominant energy source [14]. A respiratory exchange ratio > 1.1 is generally accepted as indicative of maximal exercise in adults and >1.0 in children [15]. Also, she had a low O_2_ pulse (defined as VO_2_/heart rate) of 3 mL/beat (41% of predicted) and a high ventilatory equivalent for O_2_ (as defined by VE/VO_2_) of 78 (169% of predicted), both of which are hallmarks of mitochondrial myopathy. Pre-exercise serum lactate was 2.7 mmol/L while after exercise this was 7.0 mmol/L (ref 0–2.0 mmol/L), with a ratio to pyruvate of 70. MRC enzyme assays were consequently performed demonstrating low Complex I, III and IV activity in skeletal muscle (18%, 17%, and 1% relative to citrate synthase respectively) and low Complex I and IV in liver (53% and 6% relative to citrate synthase respectively). There was no evidence of mitochondrial DNA depletion, deletion or duplications in these samples.

She performs academically well in mainstream education and participates recreationally in sailing. Systemic examination has been normal, in particular with normal tone, power and deep tendon jerk reflexes. She is post pubertal with weight on 40th percentile and height on 75th percentile. Electrolytes, liver function tests, full blood count and clotting, have been normal. At the age of 14 years, mild left ventricular posterior wall hypertrophy was identified measuring 13 mm with normal function.

### Exome sequencing

Genomic DNA was isolated from whole blood of the proband and both parents. Exome capture and library preparation was performed using the Nextera Rapid Capture kit (Illumina, San Diego, CA). Captured libraries were sequenced on an Illumina HiSeq 2000 (2 ×100 nucleotides) to a depth such that a minimum 80% of targeted bases were sequenced to a read depth of 0020× or greater. Reads were aligned to the reference human genome (GRCh37) using BWA-MEM [16], and pedigree informed variant calling was performed using the Real Time Genomics (Hamilton, New Zealand) integrated analysis tool rtgFamily v3.6.2 [17]. All variants were annotated using SnpEff v4.2 [18], SnpEff GRCh37.72 database, dbSNP138, and dbNSFP v2.9. Rare variants (MAF <0.01) were identified and assessed as previously described for their potential to disrupt protein function under different inheritance models using a custom-built interpretation interface incorporating evidence including minor allele frequency, conservation, predicted pathogenicity, and disease association [19].

### Variant validation

The *GARS* variants identified by WES analysis were confirmed by Sanger sequencing of DNA in the proband and her parents, using the ABI PRISM BigDye Terminator Cycle Sequencing Ready Reaction Kit, and ABI PRISM 3100 Genetic Analyzer (Applied Biosystems, Foster City, CA, USA).

### In silico analyses

*In silico* analyses of the *GARS* variants were performed using PolyPhen2 (http://www.genetics.bwh.harvard.edu/pph2/), SIFT (http://www.sift.jcvi.org), MutationTaster (www.mutationtaster.org/), GVGD (www.agvgd.iarc.fr/), PhyloP (www.ccg.vital-it.ch/mga/hg19/phylop), PhastCons (www.compgen.bscb.cornell.edu), ExAC (http://exac.broadinstitute.org/) and HOPE (http://www.cmbi.ru.nl/hope).

### Immunoblotting

Immunoblotting was performed as previously described [20], with the following modifications. Membranes were probed with 1:500 anti-OXPHOS (ab110411, Abcam) for 2 h at room temperature or with 1:1000 anti-GARS (ab89522, Abcam) overnight at 4°C. Protein loading was normalised to porin (1:5,000 anti-porin, ab14734, Abcam) for 2 h at room temperature. Densitometry was performed as previously described [20].

### Spectrophotometric MRC enzyme activity

MRC enzyme activities in liver and skeletal muscle biopsies from the patient were determined as previously described [21].

#### Complex I and IV dipstick enzyme activity assays in fibroblasts

Complex I and IV dipstick enzyme activities were determined as previously described [20].

### Statistical analyses

Graphpad Prism 5.03 was used for all statistical analyses. Statistical analyses were performed using the non-parametric Mann-Whitney *U* test. A *P* value less than 0.05 was considered to be statistically significant.

## Results

The presence of exercise intolerance and lactic acidosis in our patient led to an initial evaluation of MRC enzymology in skeletal muscle and liver extracts. Complex IV activity was almost undetectable in skeletal muscle, with a marked deficiency of Complex I and Complex III, suggestive of a defect in mitochondrial DNA maintenance or expression (Table 2). In the liver, Complex IV activity was markedly deficient and Complex I activity borderline low. Complex II and citrate synthase were elevated in both muscle and liver (Table 2). Additionally, we evaluated the activity of the MRC complexes I and IV in patient fibroblast extracts where we observed that the % residual activity relative to protein (% protein) were 75% (P = 0.0004) in Complex I and 45% (P < 0.0001) in Complex IV, compared to controls (Table 3). These results were consistent with the MRC enzymology results in patient skeletal muscle and liver homogenates, although the MRC deficiency was not as severe in fibroblasts.

**Table 2:** Spectrophotometric MRC enzyme diagnostic data in skeletal muscle, liver and fibroblasts. Patient liver and muscle samples. Data are expressed as activity relative to protein and as % CS ratio, which represents % of the normal control mean value when expressed relative to Citrate Synthase. Bold characters indicate clinically significant abnormal values (H–high, L–low). Complex I (CI), NADH-coenzyme Q1 oxidoreductase; Complex II (CII), succinate-coenzyme Q1 oxidoreductase; Complex III (CIII), decylbenzylquinol-cytochrome c oxidoreductase; Complex IV, cytochrome c oxidase (CIV).

	Enzyme Activity		% CS Ratio	
Muscle	Patient	(Ref Range)	Patient	(Ref Range)
CI (nmol/min/mg)	18	19–72	18 **L**	36–269
CII (nmol/min/mg)	142 **H**	26–63	126	52–156
CIII (/min/mg)	13.1	12.8–50.9	17 **L**	62–185
CIV (/min/mg)	0.21 **L**	3.3–9.1	1 **L**	36–192
CS (nmol/min/mg)	320 **H**	85–179	-	-
**Liver**				
CI (nmol/min/mg)	6	8–11	53	65–137
CII (nmol/min/mg)	116 **H**	54–73	158	59–127
CIII (/min/mg)	11.7	5.2–10.3	127	77–127
CIV (/min/mg)	0.05 **L**	0.5–0.9	6 **L**	75–134
CS (nmol/min/mg)	33	26–31	-	-

**Table 3:** Dipstick MRC enzyme data from cultured fibroblasts. Enzyme activity data are expressed as % residual activity relative to protein (% protein).

	Enzyme Activity %	
Skin fibroblasts	Patient	Control
Complex I	75% (p = 0.0004)	100%
Complex IV	45% (p < 0.0001)	100%

**Fig 1:**
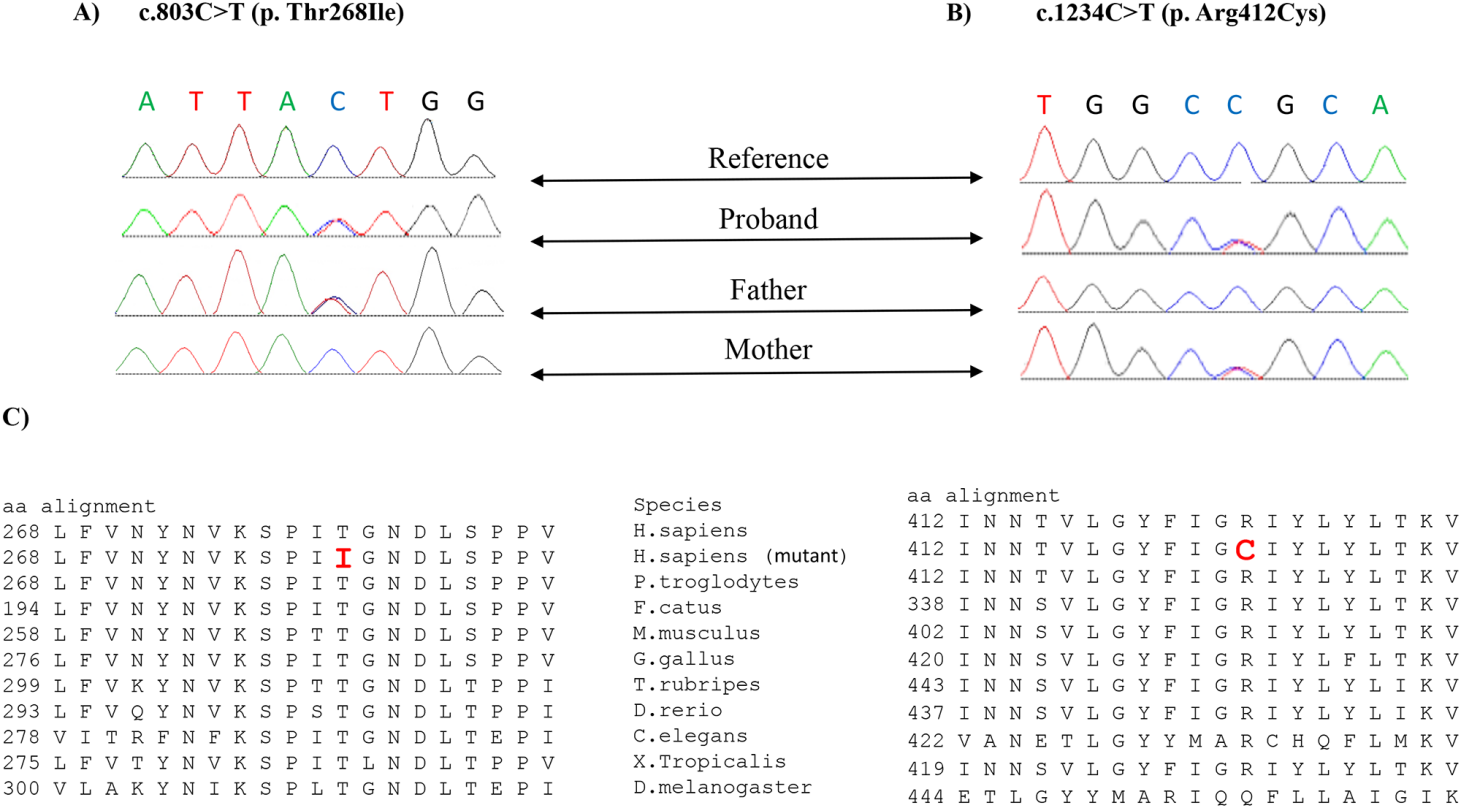
**A**) Sanger sequencing profile of *GARS* from the proband and parents showing c.803C>T; p.(Thr268Ile) variant is heterozygous in the proband and the father. **B)** Sanger sequencing profile of *GARS* from the proband and parents showing c.1234C>T; p.(Arg412Cys) variant is heterozygous in the proband and the mother. **C)** Evolutionary sequence conservations of the altered amino acid residues p.Thr268 and p. Arg412 are denoted in bold red in boxes.

Mitochondrial deletion/duplication screening was undertaken, but no structural abnormalities of the mitochondrial genome were identified. Thus, we performed whole exome sequencing and identified compound heterozygous variants, c.803C>T [p.(Thr268Ile), rs2230310] and c.1234C>T [p.(Arg412Cys), rs770924455], in *GARS* (NM_002047.3). Sanger sequencing, with each parent carrying one of the mutations (Fig 1A and 1B) confirmed both variants. Similarly, to the previous study by McMillan et al. (2014), neither parent showed symptoms or signs of CMT2D or dSMA-V, which are associated with autosomal dominant *GARS* mutations, but this has not been formally excluded by nerve conduction studies.

Both variants were considered rare with the minor allele frequency of the c.803C>T variant being 0.0031 and for the c.1234C>T variant being 0.000016 in the ExAC database (Table 4). *In silico* review of both variants (c.803C>T and c.1234C>T) predicted them to affect protein function with the most damaging score by SIFT, PolyPhen-2, Mutation-Taster and GVGD (Table 4).

**Table 4:** *In silico* analyses of the GARS variants identified in this study.

			Position		Pathogenicity prediction				Grade of conservation				
Patient	Gene	Exon	cDNA	Protein	Sift	GVGD	Polyphen-2	Mutationtster	PhyloP	PhastCons	MAF (ExAC)	Genotype	Inheritance
**Proband**	**GARS**	**7**	**c.803C>T**	**p.(Thr268Ile)**	**Damaging**	**Class C65 (most likely)**	**Probably damaging**	**disease causing**	**4.754**	**1.00**	**0.003174**	**Heterozygous**	**Paternal**
		**10**	**c.1234C>T**	**p.(Arg412Cys)**	**Damaging**	**Class C65 (most likely)**	**Probably damaging**	**disease causing**	**1.78**	**1.00**	**0.000016**	**Heterozygous**	**Maternal**

N/A: not applicable; MAF: minor allele frequency; PhyloP: values vary between -14 and +6 (Sites predicted to be conserved are assigned positive scores); PhastCons: values vary between 0 and 1. The closer the value is to 1, the more probable the nucleotide is conserve.

**Fig 2:**
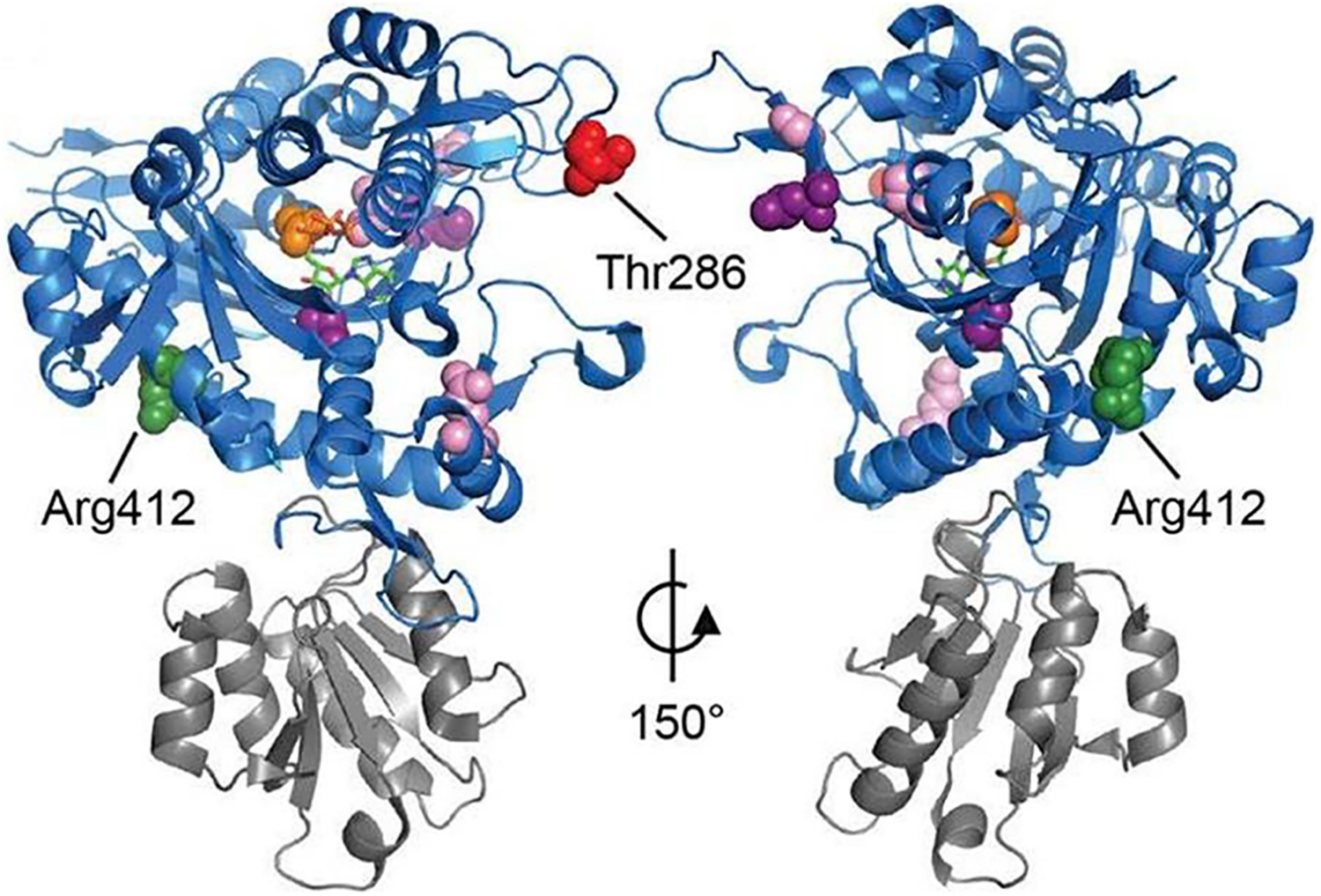
Relative position and conservation of GARS mutations. Model of GARS protein structure showing catalytic domain (blue) and anticodon binding domain (grey). Residues mutated in the proband are displayed as red (Thr268) and green (Arg412) spheres. ATP (sticks) and glycine (orange spheres) are seen in the active site pocket. Pink and purple residues indicate previously reported pathogenic mutations in CMT2D and dSMA-V respectively [6,27,28]. Model based on PDB structure 2ZT7.

Both amino acid positions (p.Thr268 and p.Arg412) are highly conserved across many species (Fig 1C). *In silico* modelling predicted the amino acid changes (p.Thr268Ile and p.Arg412Cys) to affect the overall chemical and physical properties of the *GARS* protein (http://www.cmbi.ru.nl/hope/). Both Thr268 and Arg 412 lie in the catalytic domain that synthesizes glycyl adenylate and transfers glycine to its cognate tRNA [22]. Thus, mutations in these positions are likely to disturb the protein synthesis process (http://www.cmbi.ru.nl/hope/). The wild-type residue Thr268 is smaller and less hydrophobic compared to the mutant amino acid (Ile), while the wild-type residue Arg 412 is larger, positively charged, and less hydrophobic compared to the neutral mutant amino acid, Cys (http://www.cmbi.ru.nl/hope/) (Fig 2).

**Fig 3:**
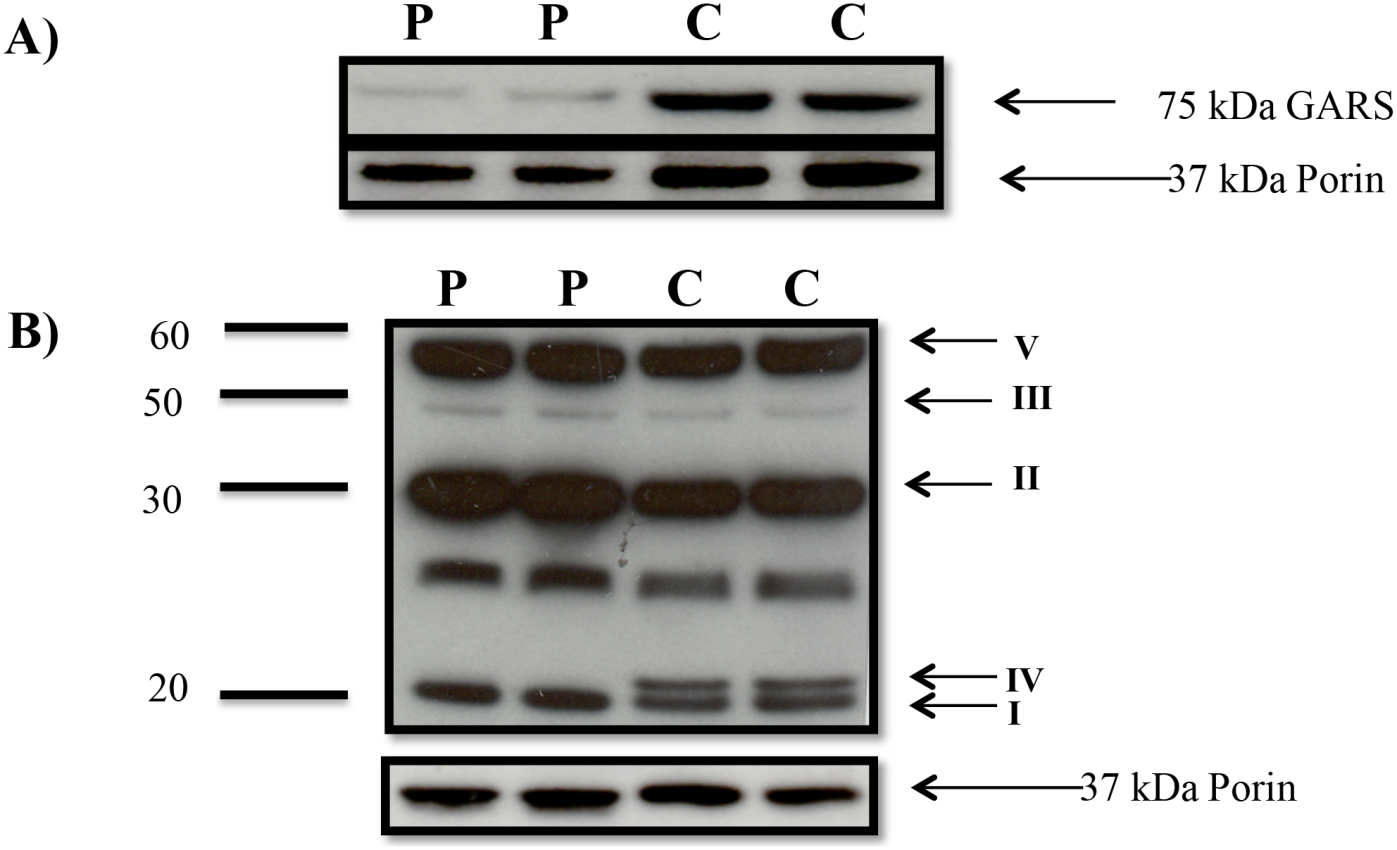
**A)** Immunoblot analysis of cultured fibroblasts lysates indicated reduction in expression of GARS protein in patient (P) compared to control (C). Samples containing 20 μg of total protein per lane were loaded in duplicate. Porin was used as a loading control. **B)** Each data point is immunoblot showing OXPHOS expression in cultured fibroblasts from the proband (P) compared to controls (C) in duplicate with a total of 30 μg of protein in each lane. Porin was used as a loading control.

Since GARS is required for synthesis of mitochondrial DNA-encoded subunits of Complex I, III, IV and V, we examined GARS protein levels and its pathogenic effect on MRC enzyme complex levels in patient fibroblast extracts. Immunoblot analysis revealed a 300% reduction in GARS protein levels in patient fibroblasts compared to a healthy control using ImageJ version 1.49 [23] (Fig 3A). Immunoblot analysis of one subunit from each MRC enzyme complex revealed undetectable levels of the mitochondrial DNA-encoded Complex IV COXII subunit in patient fibroblasts, with no reduction in the levels of the other complexes, (Fig 3B).

## Discussion

Here we report the identification of compound heterozygous *GARS* variants in a patient with exercise-intolerance, mild cardiomyopathy and lactic acidosis. *GARS* encodes both cytoplasmic and mitochondrial glycyl-tRNA synthetases which are required for protein synthesis in their respective subcellular locations [2]. Previous reports of *GARS* mutations have largely been autosomal dominant mutations associated with neuromuscular symptoms including atrophy and weakness of the hand muscles [1,5–12,24]. This phenotype closely resembles those caused by autosomal dominant mutations in cytoplasmic ARS, such as *AARS* (OMIM: 613287), *HARS* (OMIM: 616625), *YARS* (OMIM: 608323) and *MARS* (OMIM: 616280), which have been associated with distal motor neuropathy or polyneuropathies in children and adults [13]. It is thus likely that the neuropathic phenotype in autosomal dominant *GARS* is due to effects on the cytoplasmic GARS rather than mitochondrial GARS, although possible effects on mitochondrial GARS have not been investigated in these cases.

There is only one previously reported case of autosomal recessive inheritance in *GARS* where the patient displayed some clinical features similar to those seen in our patient. McMillan and colleagues reported a 12-year old girl with clinical and some biochemical features of a systemic mitochondrial disease including exercise-induced myalgia, non-compaction cardiomyopathy, persistent elevation of blood lactate, and white matter changes on brain MRI, who had compound heterozygous mutations (c.1904C>T; p.Ser635Leu and c.1787G>A; p.Arg596Gln) in *GARS* [13]. Neither patient with compound heterozygous variations in *GARS* displayed neuropathy but rather had clinical features which more closely resembled those caused by mutations in mitochondrial ARS. Mitochondrial ARS mutations are associated with a wide phenotypic spectrum, but clinical features may include leukoencephalopathy, cardiomyopathy and lactic acidosis [2].

Patients with compound heterozygous *GARS* variants also share some phenotypic overlap with patients reported to have variants in *MT-TG*, which encodes mt-tRNA^Gly^, the cognate tRNA that mitochondrial GARS attaches to glycine. *MT-TG* variants have been associated with hypertrophic cardiomyopathy and exercise intolerance [25,26].

Mitochondrial ARS mutations mainly affect mitochondrial protein synthesis [2]. Our patient displayed reduced activity of Complex I, III and IV in skeletal muscle consistent with a mitochondrial protein synthesis defect, as a number of subunits of these complexes are mitochondrially encoded. There appeared to be some tissue specific effects, with liver and fibroblasts less affected. The reduced activity of MRC complexes most likely resulted from the reduced levels and/or activity of GARS. Immunoblotting revealed a reduction in the level of the GARS protein in the patient fibroblasts, consistent with the *in silico* predictions, and suggests that the mutant GARS is less stable. Given that both variants are in the catalytic domain, it is also likely that the residual GARS have reduced activity.

Our results suggest the mitochondrial function of GARS is affected by the compound heterozygous variants we identified in the patient. While the clinical features of the McMillan case were consistent with a mitochondrial disorder, no evidence of a mitochondrial protein synthesis defect was presented and the patient had normal MRC enzyme activities in muscle [13].

The difference in GARS phenotypes observed in cases of autosomal dominant versus autosomal recessive inheritance may be a consequence of gain of function versus loss of function effects of the mutations [24]. In a mouse study, dominant mutations in *Gars* caused gain of function, with a neuropathic phenotype that could not be corrected by overexpression of wild-type *Gars*. Mice with homozygous *Gars* mutations or a missense mutation in combination with a null allele, displayed a more severe neurological phenotype resulting from loss of function [24]. In our study, we have demonstrated that the compound heterozygous *GARS* variants are also associated with loss of function. In summary, the compound heterozygous *GARS* variants identified in our patient resulted in reduced GARS protein levels and MRC enzyme deficiency. We recommend *GARS* should be added to the list of genes that should be considered in cases of exercise-intolerance and lactic acidosis.

## References

[1] Kawakami N, Komatsu K, Yamashita H, Uemura K, Oka N, Takashima H, et al (2014) [A novel mutation in glycyl-tRNA synthetase caused Charcot-Marie-Tooth disease type 2D with facial and respiratory muscle involvement]. Rinsho Shinkeigaku 54: 911–915. 25420567 10.5692/clinicalneurol.54.911

[2] Diodato D, Ghezzi D, Tiranti V (2014) The Mitochondrial Aminoacyl tRNA Synthetases: Genes and Syndromes. Int J Cell Biol 2014: 787956 10.1155/2014/787956 24639874 PMC3932222

[3] Grice SJ, Sleigh JN, Motley WW, Liu JL, Burgess RW, Talbot K, et al (2015) Dominant, toxic gain-of-function mutations in gars lead to non-cell autonomous neuropathology. Hum Mol Genet 24: 4397–4406. 10.1093/hmg/ddv176 25972375 PMC4492401

[4] Motley WW, Talbot K, Fischbeck KH (2010) GARS axonopathy: not every neuron's cup of tRNA. Trends Neurosci 33: 59–66. 10.1016/j.tins.2009.11.001 20152552 PMC2822721

[5] Liao YC, Liu YT, Tsai PC, Chang CC, Huang YH, Soong BW, et al (2015) Two Novel De Novo GARS Mutations Cause Early-Onset Axonal Charcot-Marie-Tooth Disease. PLoS One 10: e0133423 10.1371/journal.pone.0133423 26244500 PMC4526224

[6] Antonellis A, Ellsworth RE, Sambuughin N, Puls I, Abel A, Lee-Lin SQ, et al (2003) Glycyl tRNA synthetase mutations in Charcot-Marie-Tooth disease type 2D and distal spinal muscular atrophy type V. Am J Hum Genet 72: 1293–1299. 10.1086/375039 12690580 PMC1180282

[7] Sivakumar K, Kyriakides T, Puls I, Nicholson GA, Funalot B, Antonellis A, et al (2005) Phenotypic spectrum of disorders associated with glycyl-tRNA synthetase mutations. Brain 128: 2304–2314. 10.1093/brain/awh590 16014653

[8] James PA, Cader MZ, Muntoni F, Childs AM, Crow YJ, Talbot K (2006) Severe childhood SMA and axonal CMT due to anticodon binding domain mutations in the GARS gene. Neurology 67: 1710–1712. 10.1212/01.wnl.0000242619.52335.bc 17101916

[9] Rohkamm B, Reilly MM, Lochmuller H, Schlotter-Weigel B, Barisic N, Schols L, et al (2007) Further evidence for genetic heterogeneity of distal HMN type V, CMT2 with predominant hand involvement and Silver syndrome. J Neurol Sci 263: 100–106. 10.1016/j.jns.2007.06.047 17663003 PMC3272403

[10] Hamaguchi A, Ishida C, Iwasa K, Abe A, Yamada M (2010) Charcot-Marie-Tooth disease type 2D with a novel glycyl-tRNA synthetase gene (GARS) mutation. J Neurol 257: 1202–1204. 10.1007/s00415-010-5491-x 20169446

[11] Lee HJ, Park J, Nakhro K, Park JM, Hur YM, Choi BO, et al (2012) Two novel mutations of GARS in Korean families with distal hereditary motor neuropathy type V. J Peripher Nerv Syst 17: 418–421. 10.1111/j.1529-8027.2012.00442.x 23279345

[12] Klein CJ, Middha S, Duan X, Wu Y, Litchy WJ, Gu W, et al (2014) Application of whole exome sequencing in undiagnosed inherited polyneuropathies. J Neurol Neurosurg Psychiatry 85: 1265–1272. 10.1136/jnnp-2013-306740 24604904

[13] McMillan HJ, Schwartzentruber J, Smith A, Lee S, Chakraborty P, Bulman DE, et al (2014) Compound heterozygous mutations in glycyl-tRNA synthetase are a proposed cause of systemic mitochondrial disease. BMC Med Genet 15: 36 10.1186/1471-2350-15-36 24669931 PMC3973608

[14] Ten Harkel AD, Takken T, Van Osch-Gevers M, Helbing WA (2011) Normal values for cardiopulmonary exercise testing in children. Eur J Cardiovasc Prev Rehabil 18: 48–54. 10.1097/HJR.0b013e32833cca4d 20595902

[15] Arena R, Myers J, Williams MA, Gulati M, Kligfield P, Balady GJ, et al (2007) Assessment of functional capacity in clinical and research settings: a scientific statement from the American Heart Association Committee on Exercise, Rehabilitation, and Prevention of the Council on Clinical Cardiology and the Council on Cardiovascular Nursing. Circulation 116: 329–343. 10.1161/CIRCULATIONAHA.106.184461 17576872

[16] Li H, Durbin R (2009) Fast and accurate short read alignment with Burrows-Wheeler transform. Bioinformatics 25: 1754–1760. 10.1093/bioinformatics/btp324 19451168 PMC2705234

[17] Cleary JG, Braithwaite R, Gaastra K, Hilbush BS, Inglis S, Irvine SA, et al (2014) Joint variant and de novo mutation identification on pedigrees from high-throughput sequencing data. J Comput Biol 21: 405–419. 10.1089/cmb.2014.0029 24874280

[18] Cingolani P, Platts A, Wang le L, Coon M, Nguyen T, Wang L, et al (2012) A program for annotating and predicting the effects of single nucleotide polymorphisms, SnpEff: SNPs in the genome of Drosophila melanogaster strain w1118; iso-2; iso-3. Fly (Austin) 6: 80–92. 22728672 10.4161/fly.19695 PMC3679285

[19] Vanderver A, Simons C, Helman G, Crawford J, Wolf NI, Bernard G, et al (2016) Whole exome sequencing in patients with white matter abnormalities. Ann Neurol 79: 1031–1037. 10.1002/ana.24650 27159321 PMC5354169

[20] Nafisinia M, Guo Y, Dang X, Li J, Chen Y, Zhang J, et al (2016) Whole Exome Sequencing Identifies the Genetic Basis of Late-Onset Leigh Syndrome in a Patient with MRI but Little Biochemical Evidence of a Mitochondrial Disorder. JIMD Rep. 10.1007/8904_2016_541 PMC5362551 27344648

[21] Frazier AE, Thorburn DR (2012) Biochemical analyses of the electron transport chain complexes by spectrophotometry. Methods Mol Biol 837: 49–62. 10.1007/978-1-61779-504-6_4 22215540

[22] Li R, Macnamara LM, Leuchter JD, Alexander RW, Cho SS (2015) MD Simulations of tRNA and Aminoacyl-tRNA Synthetases: Dynamics, Folding, Binding, and Allostery. Int J Mol Sci 16: 15872–15902. 10.3390/ijms160715872 26184179 PMC4519929

[23] Rasband WS (1997–2016) ImageJ, U. S. National Institutes of Health, Bethesda, Maryland, USA.https://imagej.nih.gov/ij/.

[24] Motley WW, Seburn KL, Nawaz MH, Miers KE, Cheng J, Antonellis A, et al (2011) Charcot-Marie-Tooth-linked mutant GARS is toxic to peripheral neurons independent of wild-type GARS levels. PLoS Genet 7: e1002399 10.1371/journal.pgen.1002399 22144914 PMC3228828

[25] Merante F, Tein I, Benson L, Robinson BH (1994) Maternally inherited hypertrophic cardiomyopathy due to a novel T-to-C transition at nucleotide 9997 in the mitochondrial tRNA(glycine) gene. Am J Hum Genet 55: 437–446. 8079988 PMC1918404

[26] Nishigaki Y, Bonilla E, Shanske S, Gaskin DA, DiMauro S, Hirano M (2002) Exercise-induced muscle "burning," fatigue, and hyper-CKemia: mtDNA T10010C mutation in tRNA(Gly). Neurology 58: 1282–1285. 11971101 10.1212/wnl.58.8.1282

[27] Del Bo R, Locatelli F, Corti S, Scarlato M, Ghezzi S, Prelle A, et al (2006) Coexistence of CMT-2D and distal SMA-V phenotypes in an Italian family with a GARS gene mutation. Neurology 66: 752–754. 10.1212/01.wnl.0000201275.18875.ac 16534118

[28] Abe A, Hayasaka K (2009) The GARS gene is rarely mutated in Japanese patients with Charcot-Marie-Tooth neuropathy. J Hum Genet 54: 310–312. 10.1038/jhg.2009.25 19329989

